# Correction to: Molecular and pharmacological modulators of the tumor immune contexture revealed by deconvolution of RNA-seq data

**DOI:** 10.1186/s13073-019-0655-5

**Published:** 2019-07-29

**Authors:** Francesca Finotello, Clemens Mayer, Christina Plattner, Gerhard Laschober, Dietmar Rieder, Hubert Hackl, Anne Krogsdam, Zuzana Loncova, Wilfried Posch, Doris Wilflingseder, Sieghart Sopper, Marieke Ijsselsteijn, Thomas P. Brouwer, Douglas Johnson, Yaomin Xu, Yu Wang, Melinda E. Sanders, Monica V. Estrada, Paula Ericsson-Gonzalez, Pornpimol Charoentong, Justin Balko, Noel Filipe da Cunha Carvalho de Miranda, Zlatko Trajanoski

**Affiliations:** 1 Biocenter, Division of Bioinformatics, Medical University of Innsbruck, Innrain 80, Innsbruck, Austria; 2 Division of Hygiene and Medical Microbiology, Medical University of Innsbruck, Innsbruck, Austria; 3 Department of Haematology and Oncology, Medical University of Innsbruck, Innsbruck, Austria; 4 Department of Pathology, Leiden University Medical Centre, Leiden, The Netherlands; 5 Vanderbilt University, Nashville, TN USA; 6 Department of Medicine, Vanderbilt University Medical Center, Nashville, TN USA; 7 Department of Biostatistics, Vanderbilt University Medical Center, Nashville, TN USA; 8 Department Pathology Microbiology and Immunology, Vanderbilt University Medical Center, Nashville, TN USA; 9 Department of Medical Oncology and Internal Medicine VI, National Center for Tumor Diseases, University Hospital Heidelberg, Heidelberg, Germany; 10 Division of Translational Immunotherapy, German Cancer Research Center (DKFZ), Heidelberg, Germany; 11 Austrian Drug Screening Institute, Innrain 66A, Innsbruck, Austria

**Correction to: Genome Med**

**https://doi.org/10.1186/s13073-019-0638-6**

It was highlighted that the original article [1] contained a typesetting mistake in the name of Noel Filipe da Cunha Carvalho de Miranda. This was incorrectly captured as Noel Filipe da Cunha Carvahlo de Miranda. It was also highlighted that in Fig. 3c the Y-axes of the left panels were cropped and in Fig. 5c, the CD8 bar was cropped. This Correction article shows the correct Figs. 3 and 5. The original article has been updated.

**Fig. 3:**
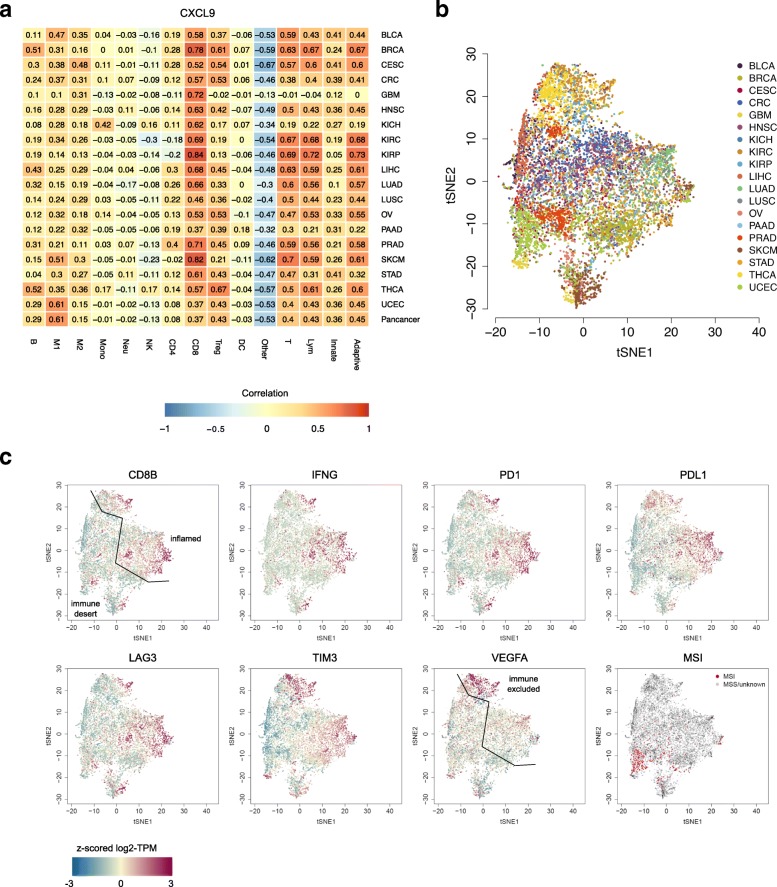
quanTIseq analysis of RNA-seq data from 19 TCGA solid cancers. **a** Pearson’s correlation between cell proportions estimated by quanTIseq and expression in TPM of the CXCL9 chemokine. t-SNE plot of the immune contextures of 8243 TCGA cancer patients, colored by: **b** cancer type or **c** expression of immune-related genes and microsatellite instability state. The line in the t-SNE plots qualitatively indicates the separation of the putative inflamed, immune-desert, and immune-excluded phenotypes. Adaptive, total adaptive immune cells; B, B cells; CD4, total CD4^+^ T cells (including also CD4^+^ regulatory T cells); CD8, CD8^+^ T cells; DC, dendritic cells; Innate, total innate immune cells; Lym, total lymphocytes; M1, classically activated macrophages; M2, alternatively activated macrophages; Mono, monocytes; MSI, microsatellite instable; MSS, microsatellite stable; Neu, neutrophils; NK, natural killer cells; Other, uncharacterized cells; T, T cells; Treg, regulatory T cells

**Fig. 5:**
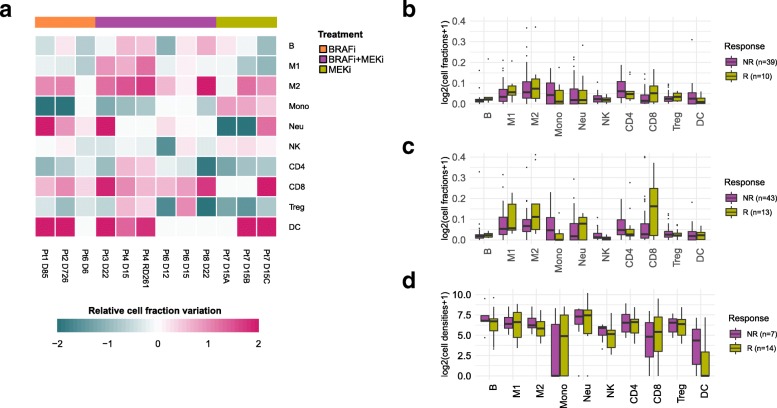
Pharmacological modulation of the tumor immune contexture and response to checkpoint blockers. **a** Changes in the immune contexture of melanoma tumors during treatment with BRAF and/or MEK inhibitors, measured as “relative cell fraction variation”, i.e., ratio between the difference and the mean of the on- and pre-treatment immune cell fractions estimated via deconvolution. Immune cell fractions (log scale) estimated with quanTIseq from pre- (**b**) and on-treatment (**c**) samples collected from melanoma patients treated with anti-PD1 and stratified as responders (R) and non-responders (NR) (data from [58]). **d** quanTIseq immune cell densities (log scale) from our cohort of melanoma patients, stratified as responders (R) and non-responders (NR). Total cell densities used to scale quanTIseq immune cell fractions were estimated as the median number of nuclei per mm^2^ across all images generated from each tumor. B, B cells; CD4, total CD4^+^ T cells (including also CD4^+^ regulatory T cells); CD8, CD8^+^ T cells; DC, dendritic cells; M1, classically activated macrophages; M2, alternatively activated macrophages; Mono, monocytes; Neu, neutrophils; NK, natural killer cells; Treg, regulatory T cells; Other, other uncharacterized cells

The Publisher apologises to the readers and the authors for any inconvenience caused.
