# Supplementary material for: Molecular and pharmacological modulators of the tumor immune contexture revealed by deconvolution of RNA-seq data
Source: Genome Med. 2019 May 24;11:34. doi: 10.1186/s13073-019-0638-6 (PMC6534875; doi:10.1186/s13073-019-0638-6)
Supplement: Supplementary file 2 — Figure S1. In silico validation of quanTIseq. Figure S2. Validation of quanTIseq on PBMC RNA-seq data from [46]. Figure S3. Validation of quanTIseq on PBMC microarray data from. Figure S4. Validation of quanTIseq on PBMC microarray data from [47]. Figure S5. QuanTIseq analysis of RNA-seq data from TCGA tumors. Figure S6. Validation of quanTIseq on nine immune cell mixtures. Figure S7. Validation of quanTIseq in solid tumors using cell densities from IF/IHC images. Figure S8. Benchmarking of IHCount on IHC images from CRC patients’ samples. Figure S9. Performance of quanTIseq and previous deconvolution methods on PBMC data. Figure S10. Correlation between quanTIseq cell fractions and genetic variables. Figure S11. Correlation between quanTIseq cell fractions and expression of chemokines and adhesion molecules. Figure S12. t-SNE plots of 8243 TCGA samples colored according to cancer type. Figure S13. t-SNE plots of 8243 TCGA samples colored according to immune cell fractions. Figure S14. Validation of quanTIseq cell densities using IHC images. Table S1. Validation data considered in this study. Table S2. Performance of quanTIseq and previous deconvolution methods. Table S3. quanTIseq parameter settings. (PDF 25199 kb) [file 13073_2019_638_MOESM2_ESM.pdf]

Supplementary Figures

Figure S1. Leave-K-out cross-validation of quanTIseq using 1,700 simulated RNA-seq data sets with various immune cell fractions and tumor contents. The cell fractions estimated via deconvolution were compared with the true read fractions and assessed with Pearson's correlation (r) and root-mean-squared error (RMSE). The true tumor contents were compared with the fractions of "other" cells estimated from quanTIseq. The line represents the linear fit. Leave-K-out cross-validation was performed by considering, for each simulated mixture to be deconvoluted, a signature matrix built excluding the K data sets included in the simulated mixture.

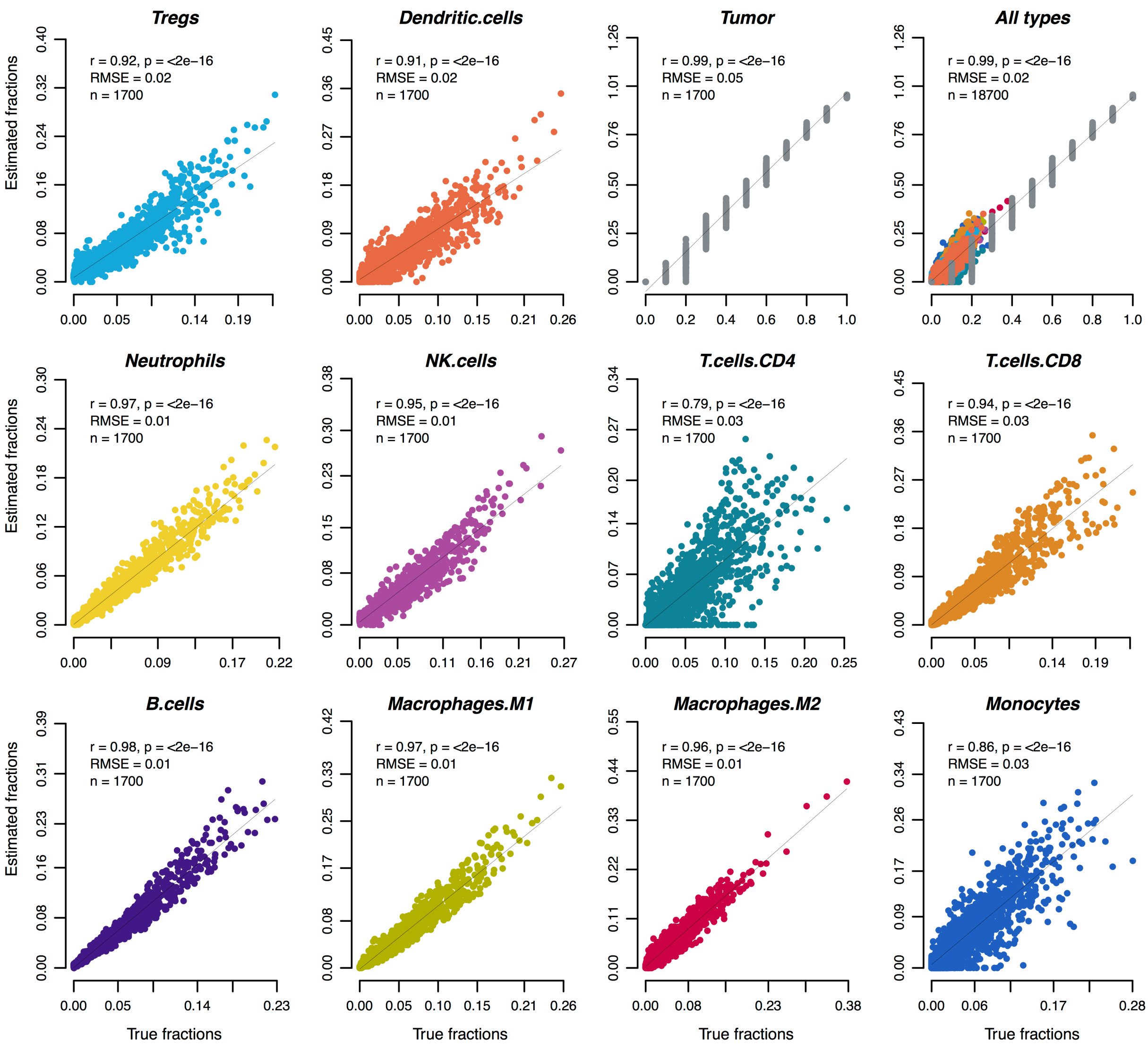

Figure S2. Validation of quanTIseq on PBMC RNA-seq data from [1]. The cell fractions estimated via deconvolution were compared with those estimated via flow cytometry and assessed with Pearson's correlation (r) and root-mean-squared error (RMSE). The grey and blue lines represent the linear fit and the "x=y" line, respectively.

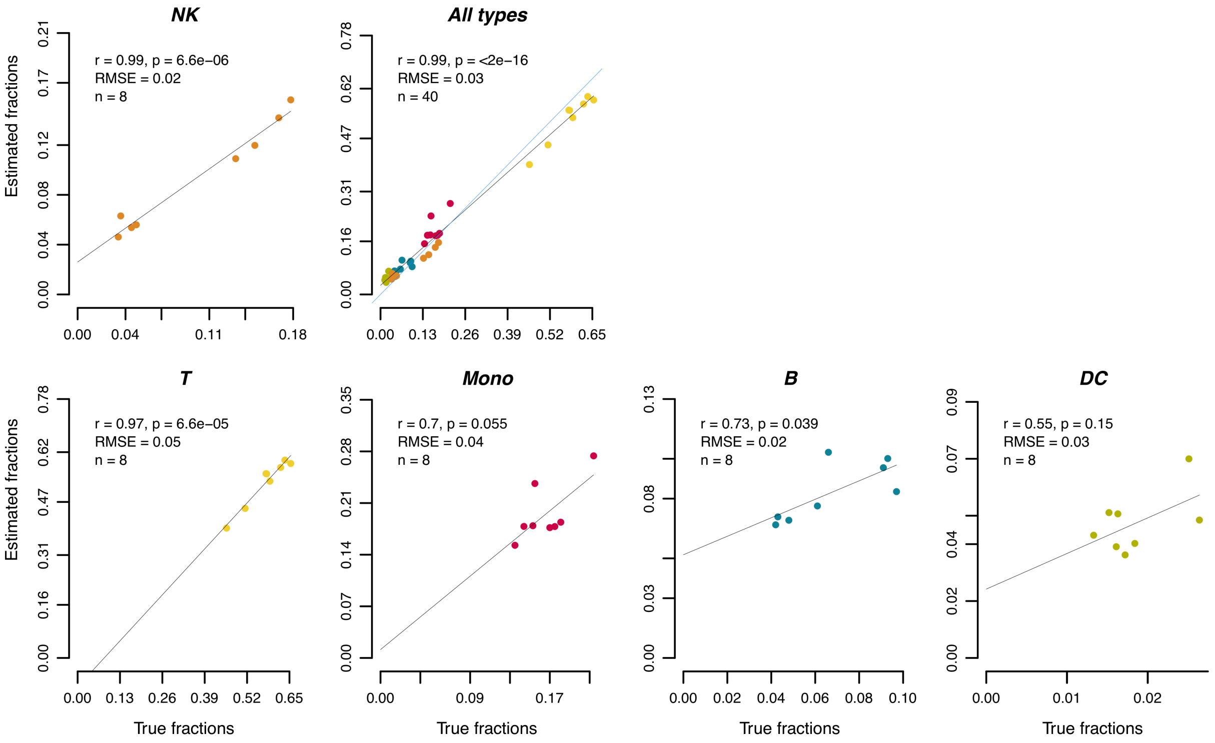

Figure S3. Validation of quanTIseq on PBMC microarray data from [2]. The cell fractions estimated via deconvolution were compared with those estimated via flow cytometry and assessed with Pearson's correlation (r) and root-mean-squared error (RMSE). The grey and blue lines represent the linear fit and the "x=y" line, respectively.

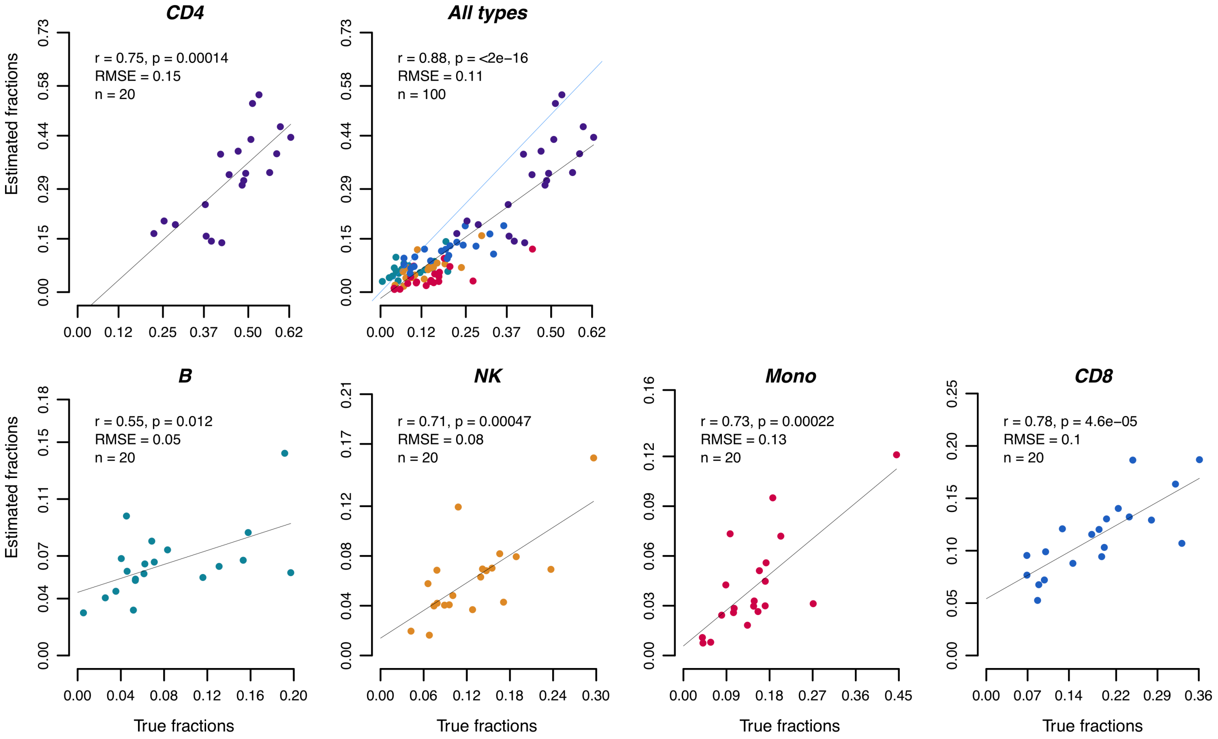

Figure S4. Validation of quanTIseq on PBMC microarray data from [3]. The cell fractions estimated via deconvolution are compared with those estimated via Coulter counter and assessed with Pearson's correlation (r) and root-mean-squared error (RMSE). The grey and blue lines represent the linear fit and the "x=y" line, respectively.

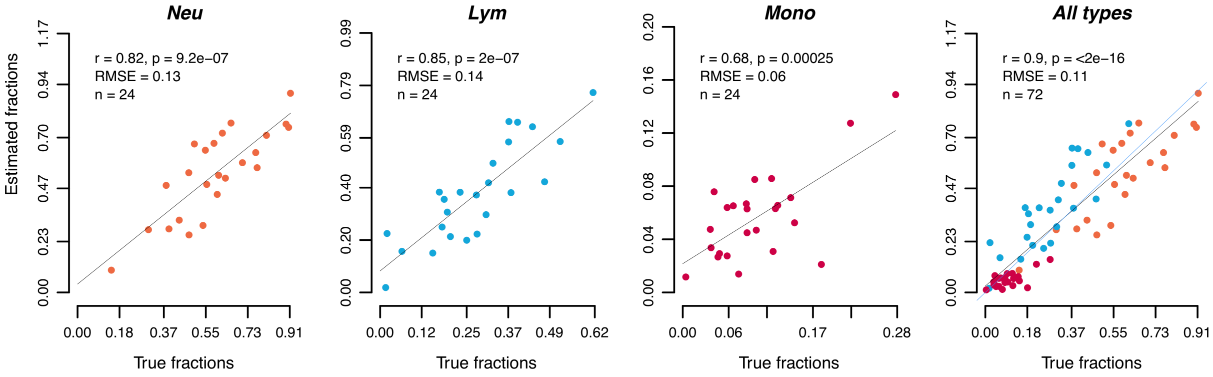

Figure S5. Analysis of RNA-seq data from TCGA tumors. **(a)** Comparison of the lymphocyte fractions computed by quanTIseq with the “Lymphocyte Scores” estimated from digital pathology images of melanoma tumors in [4]. The p-vale of the Kruskal-Wallis test and the area under the receiver operating characteristic curve (AUROC), computed for all the six score levels and for the two most extreme ones, are reported**. (b)** Comparison of the fraction of “other” cells computed by quanTIseq with the tumor purity values estimated in [5]. The agreement was assessed with Pearson’s correlation (r) and root-mean-squared error (RMSE). The line represents the linear fit.

a

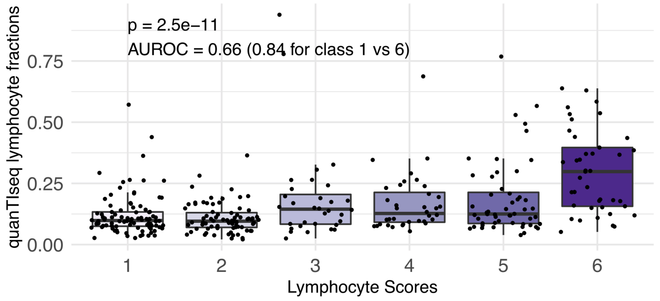

b

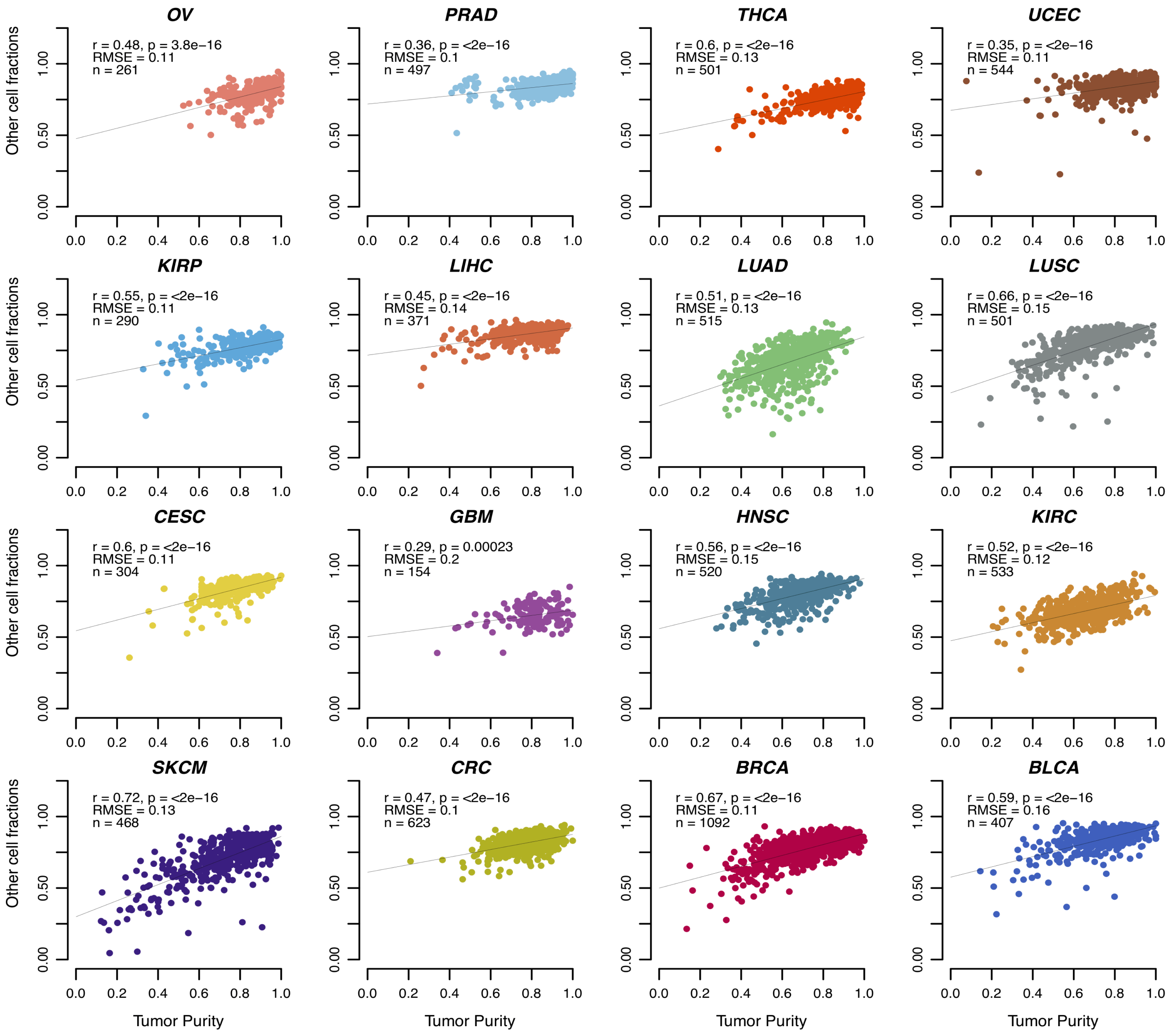

Figure S6. Validation of quanTIseq on nine immune-cell mixtures profiled with RNA-seq data within this study. The cell fractions estimated via deconvolution were compared with those estimated via flow cytometry and assessed with Pearson's correlation (r) and root-mean-squared error (RMSE). The grey and blue lines represent the linear fit and the "x=y" line, respectively.

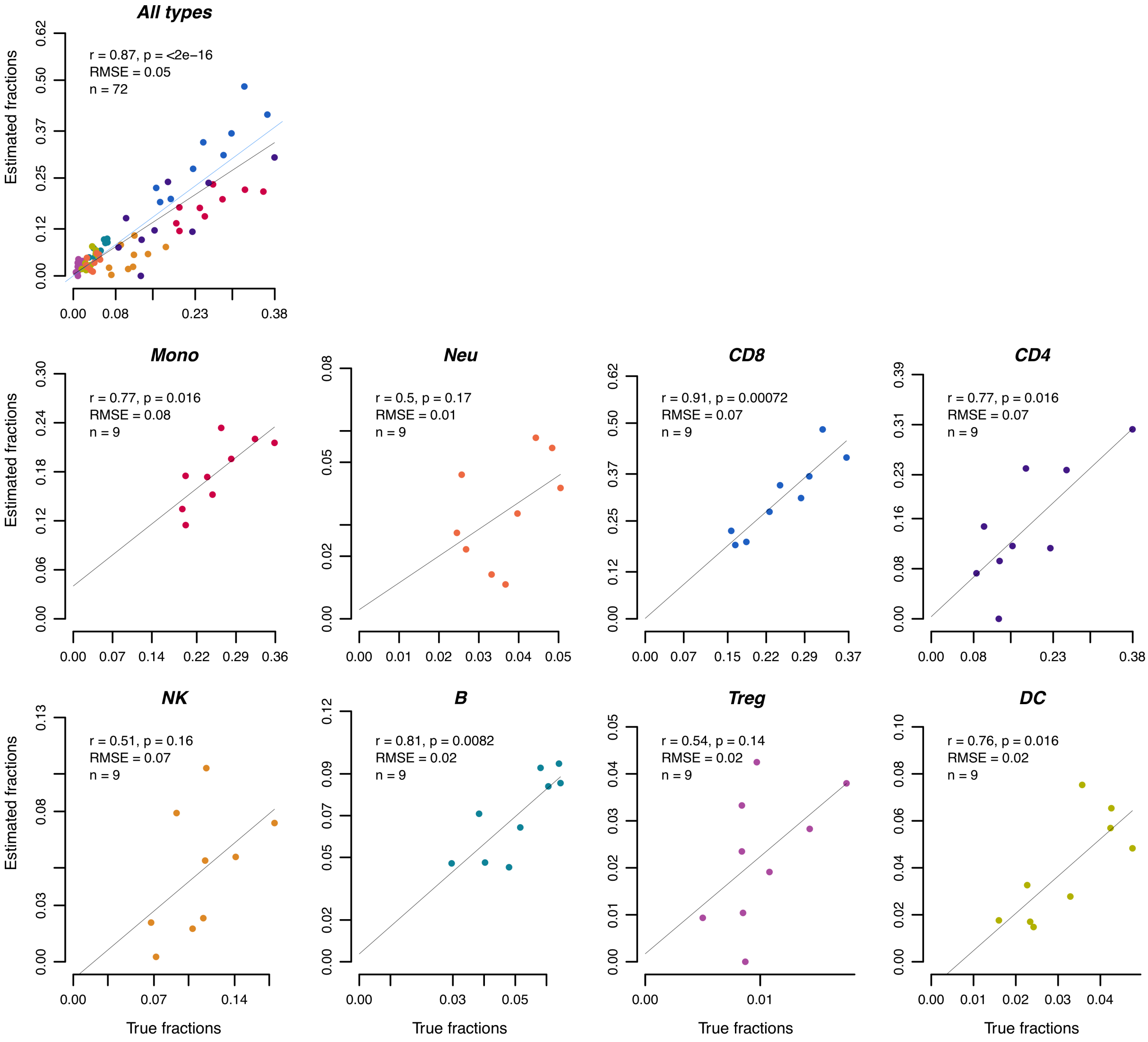

Figure S7. Comparison of quanTIseq cell fractions with cell densities inferred from IF/IHC images from melanoma **(a)**, lung cancer **(b)**, and colorectal cancer **(c)** patients. Deconvolution performance was assessed with Pearson's correlation (r) considering the image cell densities as ground truth. The line represents the linear fit. B: B cells. CD4: total $CD4^+$ T cells (including also $CD4^+$ regulatory T cells); CD8: $CD8^+$ T cells; M2: M2 macrophages; T: Treg: regulatory T cells.

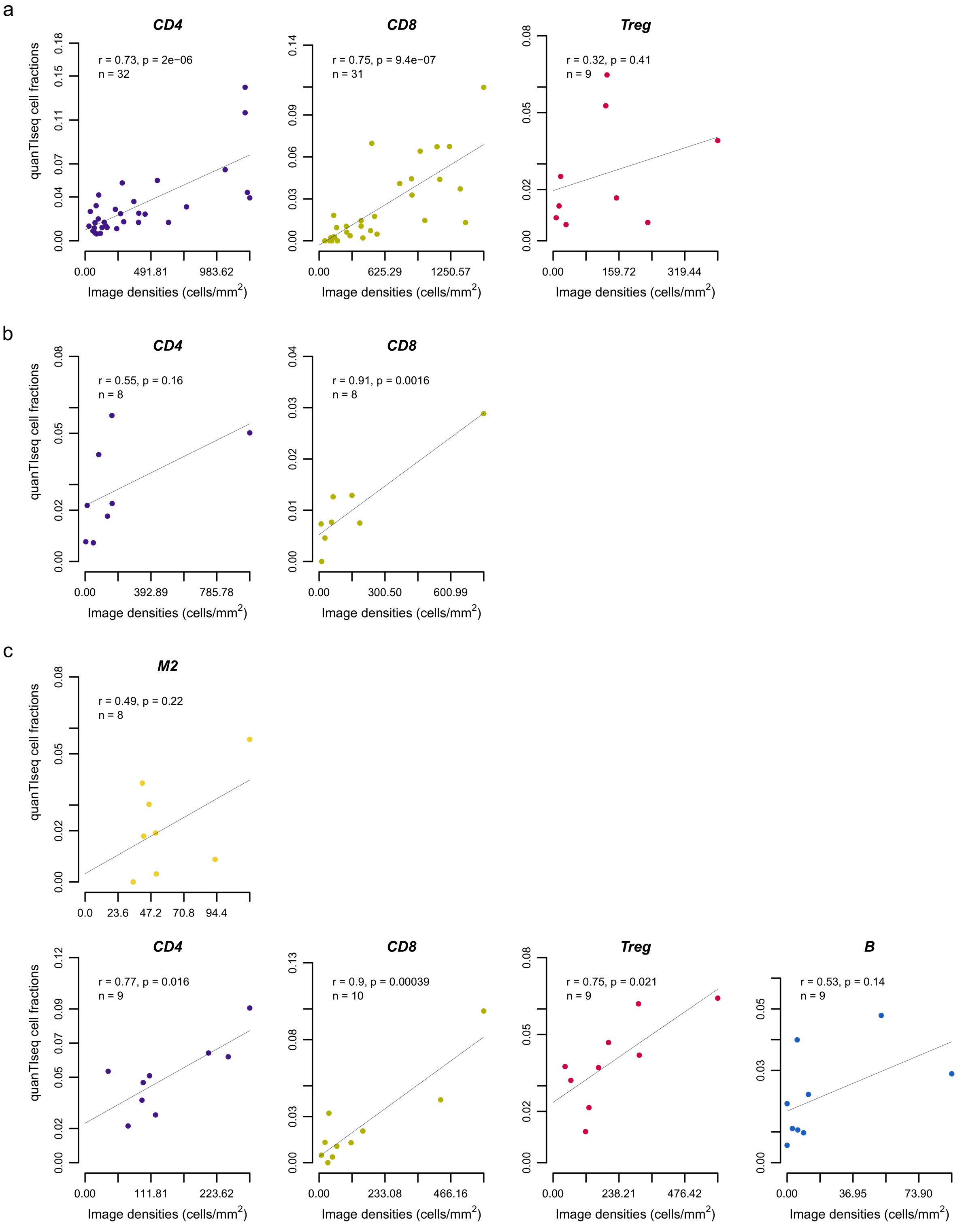

Figure S8. Benchmarking of IHCount on IHC images from CRC patients' samples. IHCount cell fractions **(a)** and densities **(b)** were compared to the gold standard estimates obtained using Perkin Elmer (http://www.perkinelmer.com) proprietary software for automated quantitative pathology, also used for quanTIseq validation. The agreement was assessed with Pearson's correlation (r) and root-mean-squared error (RMSE). The grey and blue lines represent the linear fit and the "x=y" line, respectively.

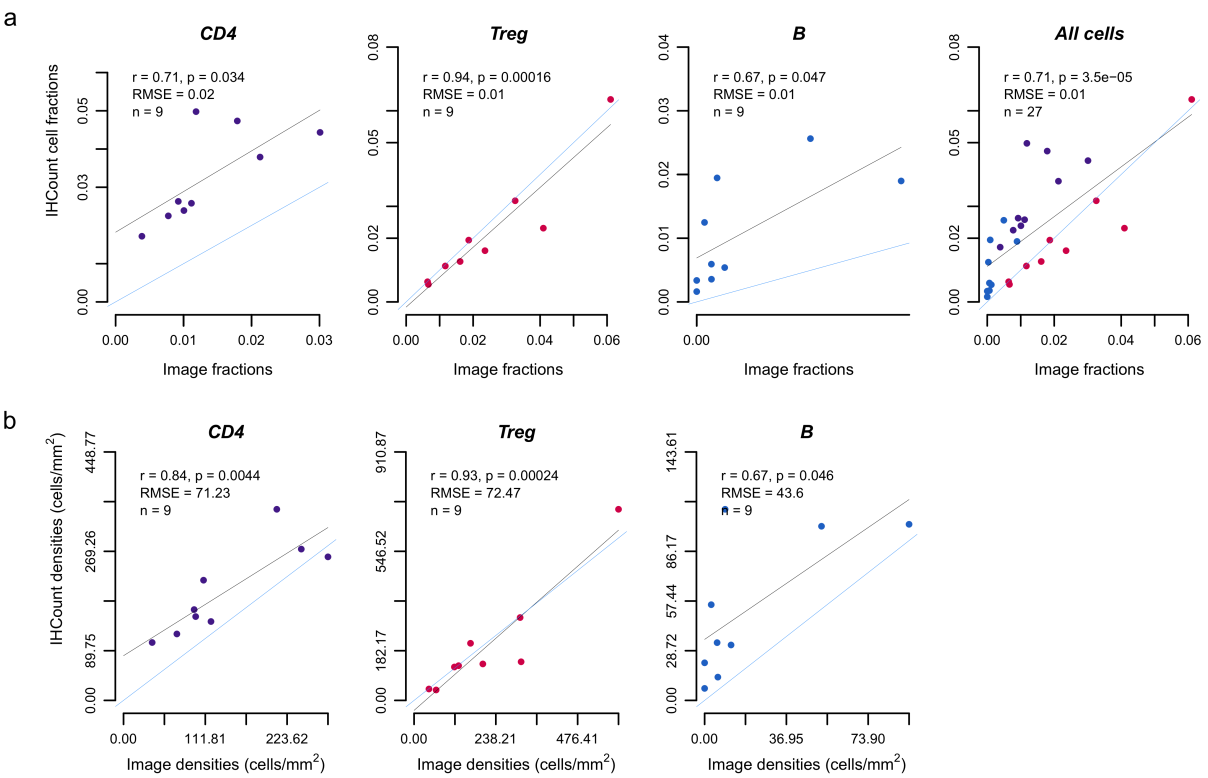

Figure S9. Performance of quanTIseq and previous deconvolution methods obtained on PBMC data generated in [1] **(c)** and in this study **(b)**. Methods performance was quantified using Pearson's correlation (r) considering the flow cytometry estimates as ground truth. Correlations for single cell types and are displayed as single dots, together with whiskers and horizontal bands representing median and 95% confidence intervals. Missing cell types are visualized as triangles at the bottom of the plots. Diamonds indicate the overall correlation obtained considering all cell types together; not shown for marker-based methods, which do not allow intra-sample comparison.

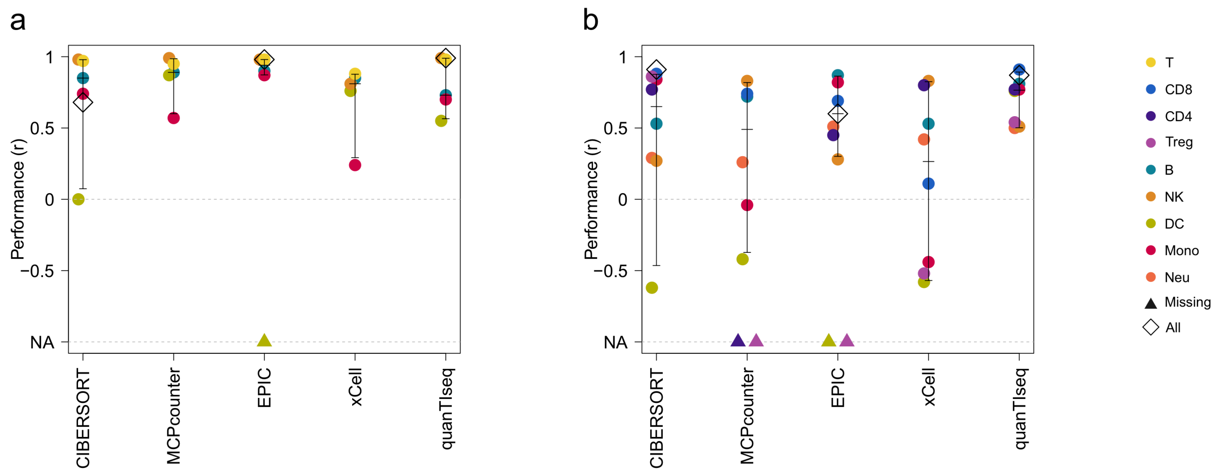

Figure S10. Pearson's correlation between the cell fractions estimated by quanTIseq and the mutational load (i.e. $\log_{10}$ non-synonymous mutations, **(a)**, $\log_{10}$ non-synonymous mutations per megabase **(b)**, $\log_{10}$ neoantigens **(c)**, tumor heterogeneity measured as the area under the curve (AUC) of the cumulative density function from all cancer cell fractions **(d)**, and fractions of clonal **(e)** and subclonal mutations **(f)**. Correlations were computed for cancer types having paired correlates for at least 50 patients.

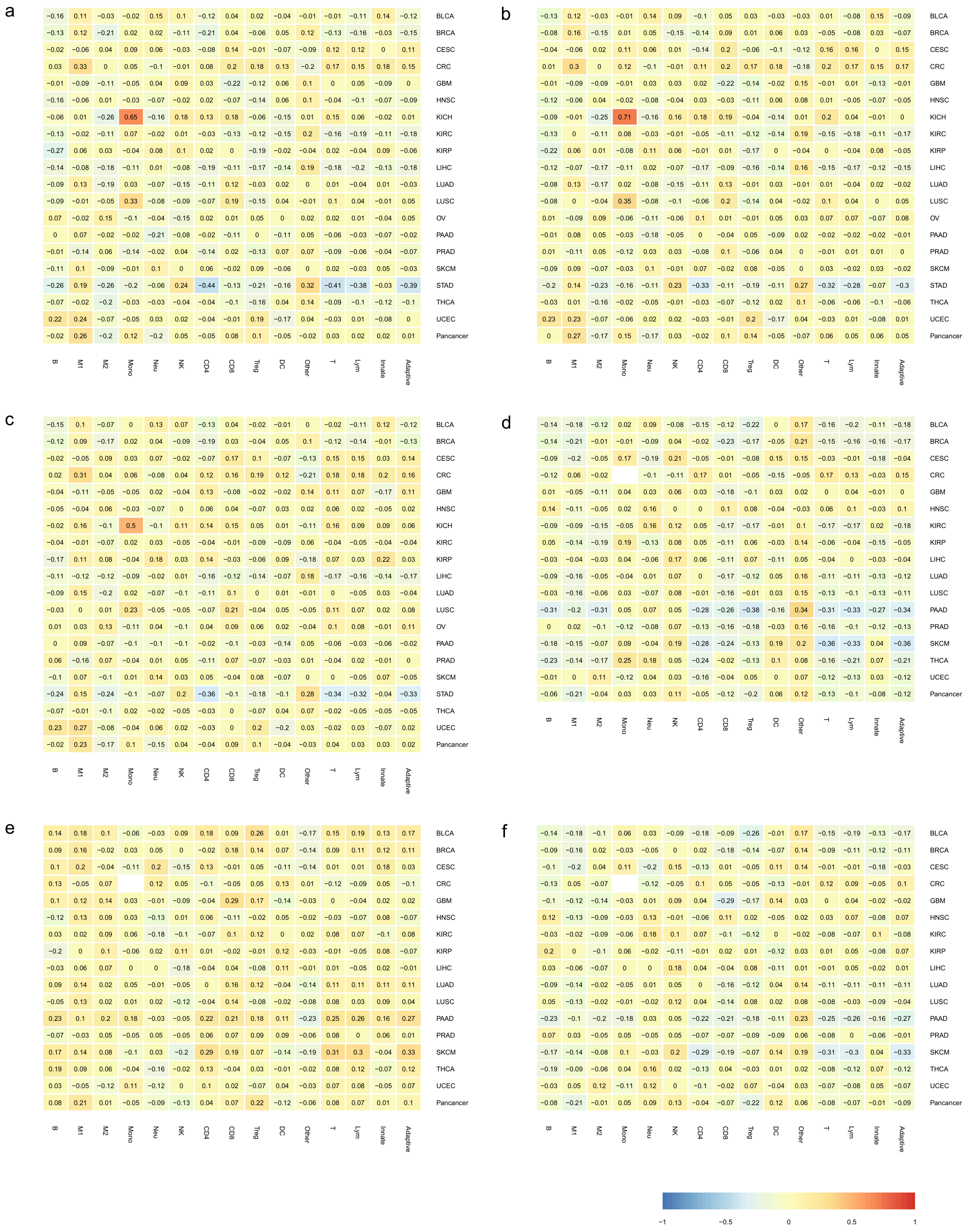

Figure S11. Pearson's correlation between the cell fractions estimated by quanTIseq and the expression in TPM of CXCL10 **(a)**, CXCR3 **(b)**, CX3CL1 **(c)**, ICAM1 **(d)**, VCAM1 **(e)**, and MADCAM1 **(f)**.

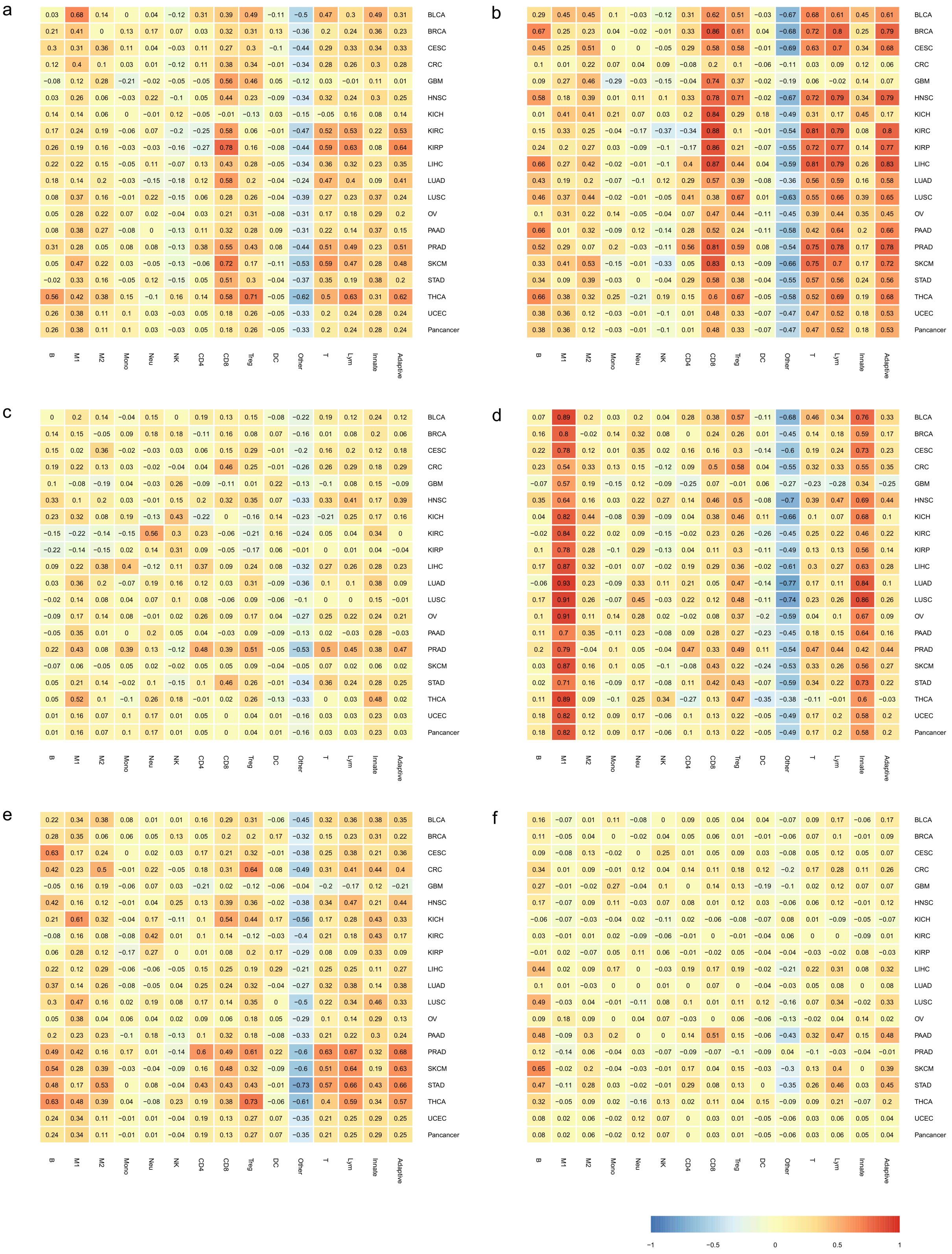

Figure S12. t-SNE plots of the immune contexture across 8,243 TCGA cancer patients (corresponding to **Figure 3b** in the main text), colored according to cancer type.

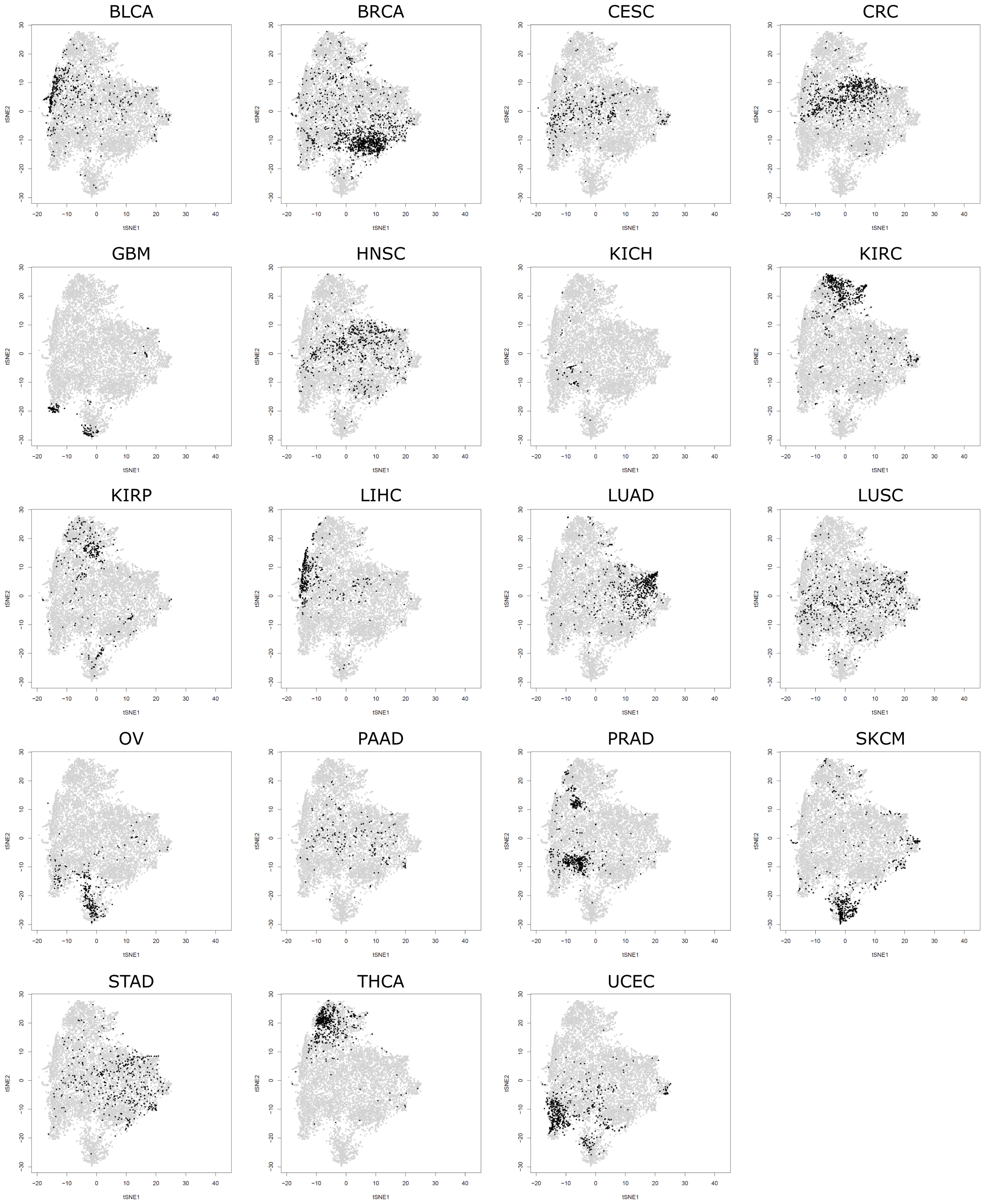

Figure S13. t-SNE plots of the immune contexture across 8,243 TCGA cancer patients (corresponding to **Figure 3b** in the main text) colored according to the deconvoluted immune cell fractions. B: B cells; CD4: total CD4^{+} T cells (including also CD4^{+} regulatory T cells); CD8: CD8^{+} T cells; DC: dendritic cells; M1: classically activated macrophages; M2: alternatively activated macrophages; Mono: monocytes; Neu: neutrophils; NK: natural killer cells; Treg: regulatory T cells.

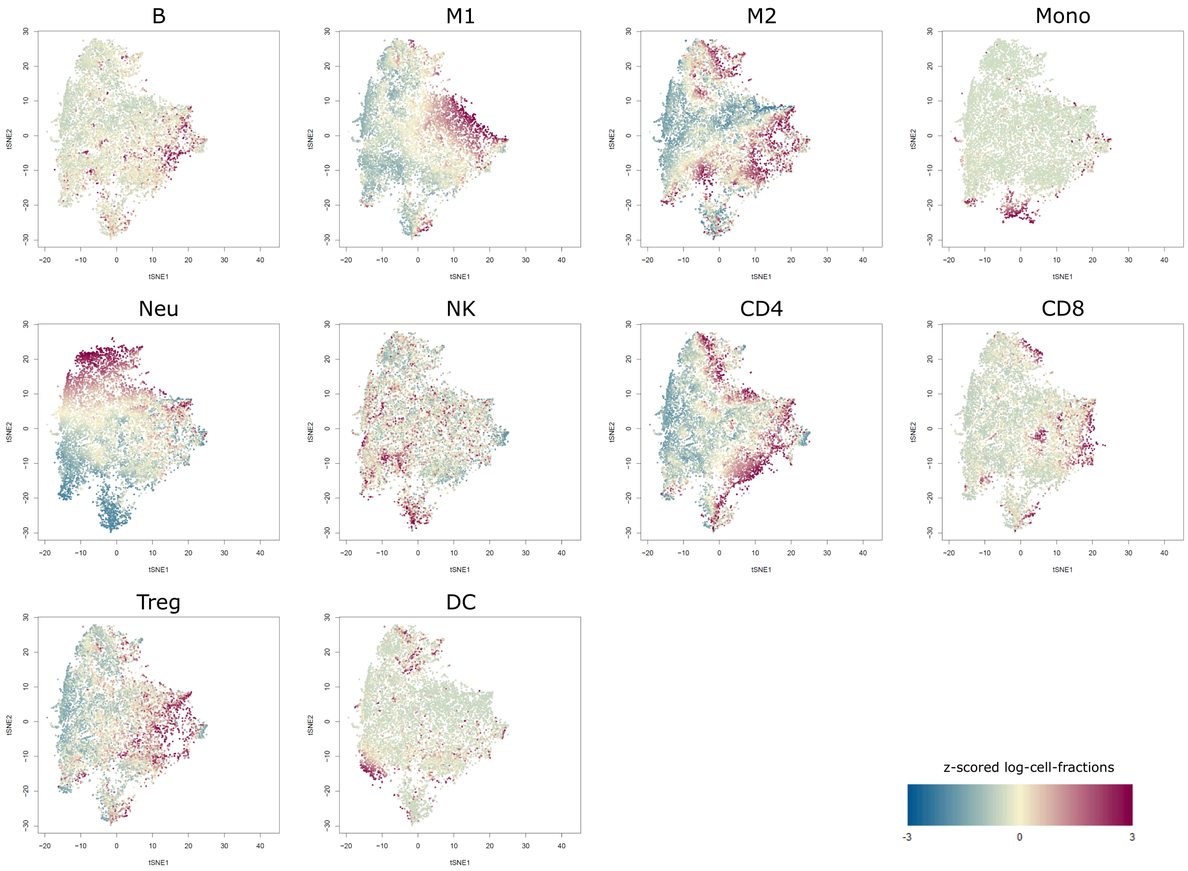

Figure S14. Comparison of quanTIseq cell densities with IHC-derived cell densities estimated from melanoma samples and assessment of Pearson's correlation (r) and root-mean-squared error (RMSE). The line represents the linear fit. CD4: total CD4$^+$ T cells (including also CD4$^+$ regulatory T cells); CD8: CD8$^+$ T cells; T: Treg: regulatory T cells.

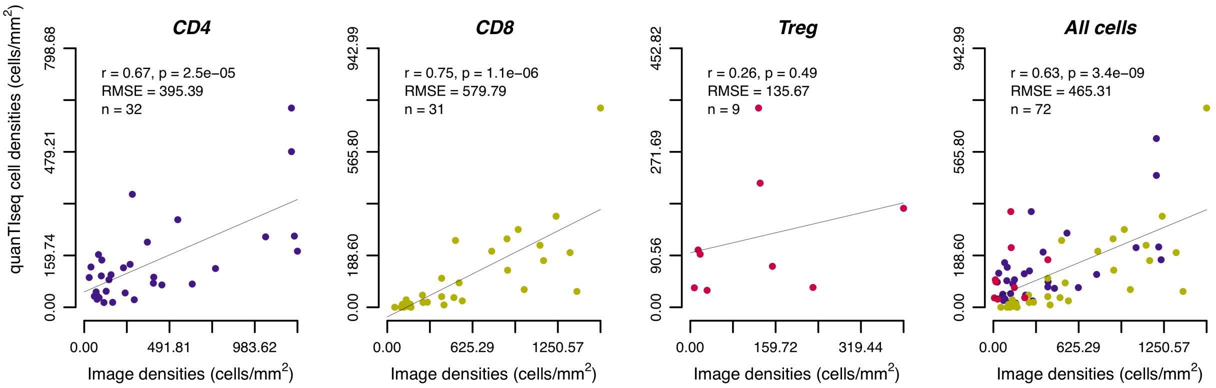

Supplementary Tables

Table S1. Validation data considered in this study: data set ID, sample type and size, availability of expression data, availability of validation data, reference publication.

ID	Samples	Expression data	Other data	Ref.
**1**	100 simulated breast tumors infiltrated with immune cells.	Gene expression as TPM and read counts (http://icbi.at/quantiseq).	mRNA fractions for: B cells, M1 and M2 macrophages, monocytes, neutrophils, NK cells, CD4$^+$ and CD8$^+$ T cells, T_{reg} cells, and dendritic cells (http://icbi.at/quantiseq).	This study
**2**	PBMC from 8 healthy subjects after influenza vaccination.	FASTQ files from Illumina RNA-seq (SRA: SRP051688).	Flow cytometry cell fractions for: T cells, B cells, NK cells, myeloid dendritic cells, monocytes, and neutrophils (data personally communicated by the authors).	[1]
**3**	PBMC from 20 healthy individuals receiving influenza immunization.	Normalized gene expression from Illumina HumanHT-12 V4.0 expression beadchip (GEO: GSE65133).	Flow cytometry cell fractions for: naïve B cells, memory B cells, CD8$^+$ T cells, naïve CD4$^+$ T cells, resting memory CD4$^+$ T cells, activated memory CD4$^+$ T cells, Gamma-delta T cells, NK cells, monocytes (GEO accession: GSE65133).	[2]
**4**	Blood from 24 pediatric renal transplant patients.	Normalized gene expression from Affymetrix Human Genome U133 Plus 2.0 Array (GEO: GSE20300).	Coulter counter fractions for neutrophils, lymphocytes, monocytes, eosinophils, basophils (supplementary material in the original publication).	[3]
**5**	8,243 solid tumors from 20 TCGA cancer types.	TPM expression values from Illumina RNA-seq (TCGA Data Portal, https://tcga-data.nci.nih.gov/tcga).	Lymphocyte scores computed from H&E-stained section slides[4]; tumor purity values estimated from NGS data[5]; survival data (http://firebrowse.org); microsatellite instability (MSI) state[6]; mutational load, neoantigen load, tumor heterogeneity, number of clonal and subclonal mutations (tcia.eu)[7].	[8]
**6**	Mixtures of PBMC and PMN cells from 9 healthy donors.	FASTQ files from Illumina RNA-seq (GEO: GSE107572).	Flow cytometry cell fractions for: B cells, monocytes, neutrophils, NK cells, CD4$^+$ and CD8$^+$ T cells, T_{reg} cells, and dendritic cells (will be available upon publication)	This study
**7**	70 tumor samples from melanoma patients treated with checkpoint blockers.	FASTQ files from Illumina RNA-seq (data personally provided by the authors).	Images of IHC slides stained for one or more of the following markers: CD8, CD4, and FOXP3.	[9]
**8**	Tumor samples from 8 lung cancer patients treated with checkpoint blockers.	FASTQ files from Illumina RNA-seq (data personally provided by the authors)	Images of IHC slides stained for one or more of the following markers: CD8, CD4, and FOXP3.	This study
**9**	Fresh-frozen tumor specimens from 10 colorectal cancer patients.	FASTQ files from Illumina RNA-seq (data personally provided by the authors).	Cell fractions and densities estimated from IF (CD8, CD68/HLA-DR/CD163) or IHC slides (CD4, FOXP3, CD20).	This study
**10**	Tumor biopsies from 7 melanoma patients collected before and during treatment with BRAF and/or MEK inhibitors.	FASTQ files from Illumina RNA-seq (SRA: SRP066571).		[10]
**11**	Tumor biopsies (51 pre- and 58 on-treatment) from 65 melanoma patients treated with checkpoint blockers.	FASTQ files from Illumina RNA-seq (SRA: SRP094781).	Response to therapy available from (GEO: GSE91061).	[11]

Table S2. Summary of the performance of quanTIseq and previous deconvolution methods obtained on the validation data sets and measured as Pearson's correlation (r). NAs indicate that all the estimated cell fractions were null, whereas dashes indicate absent or non-meaningful results (i.e., overall correlation across all cell types for methods that do not allow intra-sample comparison, analysis of PBMC data with TIMER). Data set IDs refer to **Table S1**.

ID	Cell type	CIBERSORT	MCPcounter	EPIC	xCell	TIMER	quanTIseq
**2**	All cell types	0.68	-	0.98	-	-	0.99
**2**	B cells	0.85	0.89	0.90	0.85	-	0.73
**2**	Dendritic cells	NA	0.87	-	0.76	-	0.55
**2**	Monocytes	0.74	0.57	0.87	0.24	-	0.70
**2**	NK cells	0.98	0.99	0.98	0.81	-	0.99
**2**	T cells	0.97	0.95	0.98	0.88	-	0.98
**6**	All cell types	0.91	-	0.60	-	-	0.87
**6**	B cells	0.53	0.72	0.87	0.53	-	0.81
**6**	Dendritic cells	-0.62	-0.42	-	-0.58	-	0.76
**6**	Monocytes	0.84	-0.04	0.82	-0.44	-	0.77
**6**	Neutrophils	0.29	0.26	0.51	0.42	-	0.50
**6**	NK cells	0.27	0.83	0.28	0.83	-	0.51
**6**	T cells CD4	0.77	-	0.45	0.80	-	0.77
**6**	T cells CD8	0.88	0.74	0.69	0.11	-	0.91
**6**	Tregs	0.86	-	-	-0.52	-	0.54
**7**	T cells CD4	0.67	-	0.62	0.60	0.62	0.52
**7**	T cells CD8	0.52	0.74	0.16	0.77	-0.10	0.75
**7**	Tregs	-0.20	-	-	0.45	-	0.32
**8**	T cells CD4	0.66	-	0.48	0.31	0.38	0.58
**8**	T cells CD8	0.71	0.05	-0.29	0.67	0.78	0.91
**9**	B cells	0.8	0.61	0.54	0.37	0.26	0.52
**9**	M2 macrophages	0.75	-	-	0.28	-	0.56
**9**	T cells CD4	-0.32	-	0.30	-0.17	0.00	0.80
**9**	T cells CD8	0.88	0.65	0.32	0.72	0.69	0.86
**9**	Tregs	-0.26	-	-	0.22	-	0.74

Table S3. quanTIseq parameter settings used for the analysis of the validation data. Data set IDs refer to **Table S1**.

Dataset ID	tumor	arrays	rmgenes	mRNAscale	method
**1**	TRUE	FALSE	default	FALSE	lsei
**2**	FALSE	FALSE	default	TRUE	lsei
**3**	FALSE	TRUE	none	TRUE	lsei
**4**	FALSE	TRUE	none	TRUE	lsei
**5**	TRUE	FALSE	default	TRUE	lsei
**6**	FALSE	FALSE	default	TRUE	lsei
**7**	TRUE	FALSE	default	TRUE	lsei
**8**	TRUE	FALSE	default	TRUE	lsei
**9**	TRUE	FALSE	default	TRUE	lsei
**10**	TRUE	FALSE	default	TRUE	lsei
**11**	TRUE	FALSE	default	TRUE	lsei

References

1. Hoek KL, Samir P, Howard LM, Niu X, Prasad N, Galassie A, et al. A cell-based systems biology assessment of human blood to monitor immune responses after influenza vaccination. PloS One. 2015;10:e0118528.

2. Newman AM, Liu CL, Green MR, Gentles AJ, Feng W, Xu Y, et al. Robust enumeration of cell subsets from tissue expression profiles. Nat Methods. 2015;12:453–7.

3. Shen-Orr SS, Tibshirani R, Khatri P, Bodian DL, Staedtler F, Perry NM, et al. Cell type-specific gene expression differences in complex tissues. Nat Methods. 2010;7:287–9.

4. Cancer Genome Atlas Network. Genomic Classification of Cutaneous Melanoma. Cell. 2015;161:1681–96.

5. Aran D, Sirota M, Butte AJ. Systematic pan-cancer analysis of tumour purity. Nat Commun. 2015;6:8971.

6. Cortes-Ciriano I, Lee S, Park W-Y, Kim T-M, Park PJ. A molecular portrait of microsatellite instability across multiple cancers. Nat Commun. 2017;8:15180.

7. Charoentong P, Finotello F, Angelova M, Mayer C, Efremova M, Rieder D, et al. Pan-cancer Immunogenomic Analyses Reveal Genotype-Immunophenotype Relationships and Predictors of Response to Checkpoint Blockade. Cell Rep. 2017;18:248–62.

8. Cancer Genome Atlas Research Network, Weinstein JN, Collisson EA, Mills GB, Shaw KRM, Ozenberger BA, et al. The Cancer Genome Atlas Pan-Cancer analysis project. Nat Genet. 2013;45:1113–20.

9. Johnson DB, Estrada MV, Salgado R, Sanchez V, Doxie DB, Opalenik SR, et al. Melanoma-specific MHC-II expression represents a tumour-autonomous phenotype and predicts response to anti-PD-1/PD-L1 therapy. Nat Commun. 2016;7:10582.

10. Song C, Piva M, Sun L, Hong A, Moriceau G, Kong X, et al. Recurrent Tumor Cell-Intrinsic and -Extrinsic Alterations during MAPKi-Induced Melanoma Regression and Early Adaptation. Cancer Discov. 2017;7:1248–65.

11. Riaz N, Havel JJ, Makarov V, Desrichard A, Urba WJ, Sims JS, et al. Tumor and Microenvironment Evolution during Immunotherapy with Nivolumab. Cell. 2017;171:934–49.e16.
